# Supplementary material for: Soil Ca2SiO4 Supplying Increases Drought Tolerance of Young Arabica Coffee Plants
Source: Plants (Basel). 2025 Dec 2;14(23):3666. doi: 10.3390/plants14233666 (PMC12694557; doi:10.3390/plants14233666)
Supplement: Supplementary file 1 [file plants-14-03666-s001.zip › plants-3989922-supplementary.pdf]

plants

MDPI

Supplementary Materials for Article

Soil Ca_2SiO_4 Supplying Increases Drought Tolerance of Young Arabica Coffee Plants

Miroslava Rakocevic [1,2,*], Rafael Vasconcelos Ribeiro [1]

[1] Laboratory of Crop Physiology, Department of Plant Biology, Institute of Biology, State University of Campinas (UNICAMP), Campinas 13083-862, SP, Brazil; mima.rakocevic61@gmail.com; rvr@unicamp.br

[2] Laboratório de Melhoramento Genético Vegetal, Centro de Ciências e Tecnologias Agropecuárias, Setor de Fisiologia Vegetal, Universidade Estadual do Norte Fluminense, Avenida Alberto Lamego, 2000, Parque Califórnia, Campos dos Goytacazes 28013-602, RJ, Brazil.

* Correspondence: mima.rakocevic61@gmail.com; Tel.: 55-19-97161-8918

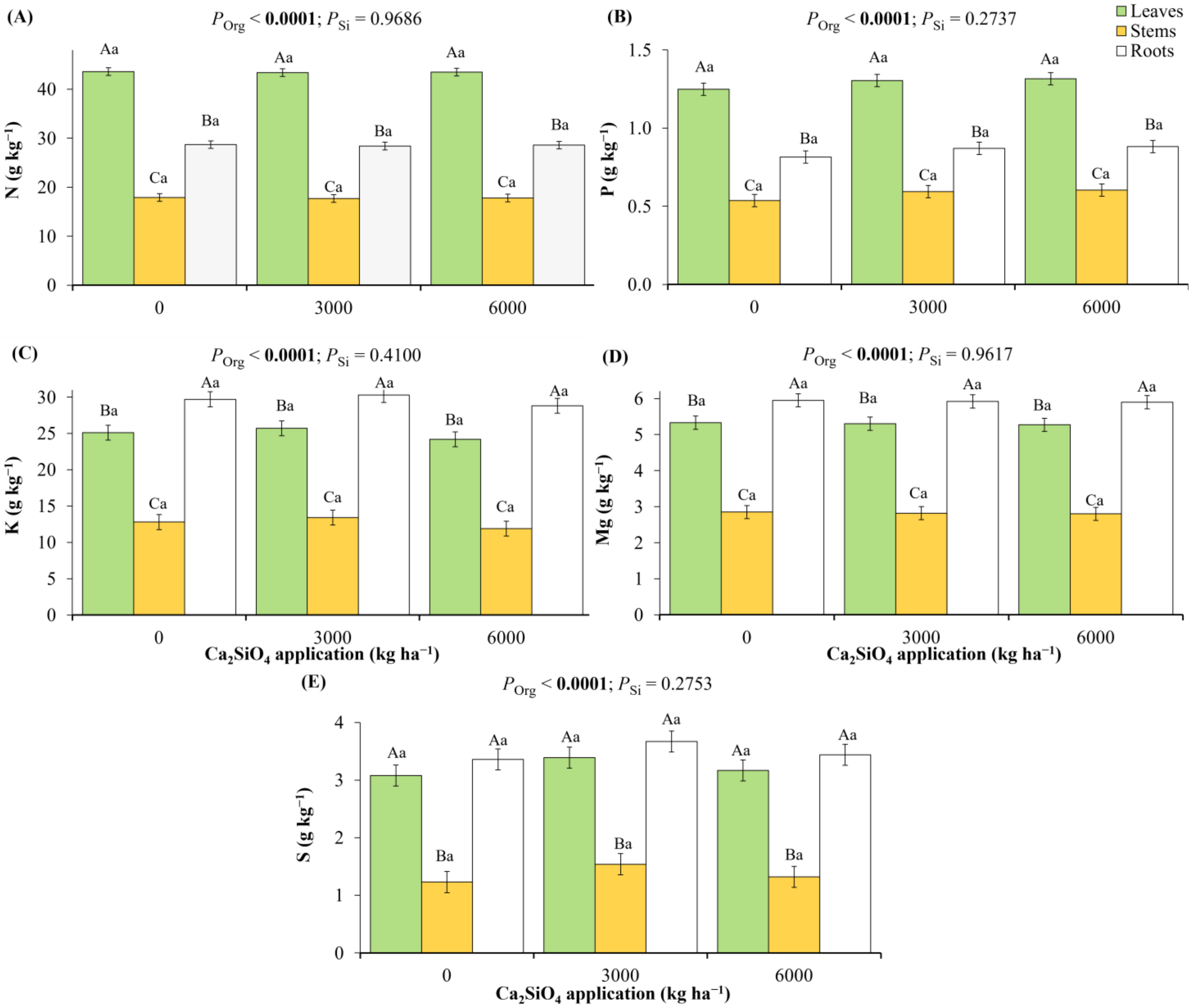

Figure S1. Variations in macronutrients: **A**) N, **B**) P, **C**) K, **D**) Mg, and **E**) S in leaves, stems, and roots of young coffee plants grown for 130 days under Ca_2SiO_4 supplying, corresponding to 0, 3000, and 6000 kg ha^{-1}. Estimated mean± SE, and *P*-values (bold when

significant) are shown (n=3). Uppercase letters compare macronutrient concentrations among organs (Org) for each Ca_2SiO_4 treatment, while lowercase letters compare concentrations among Ca_2SiO_4 treatments (Si) for each plant organ.

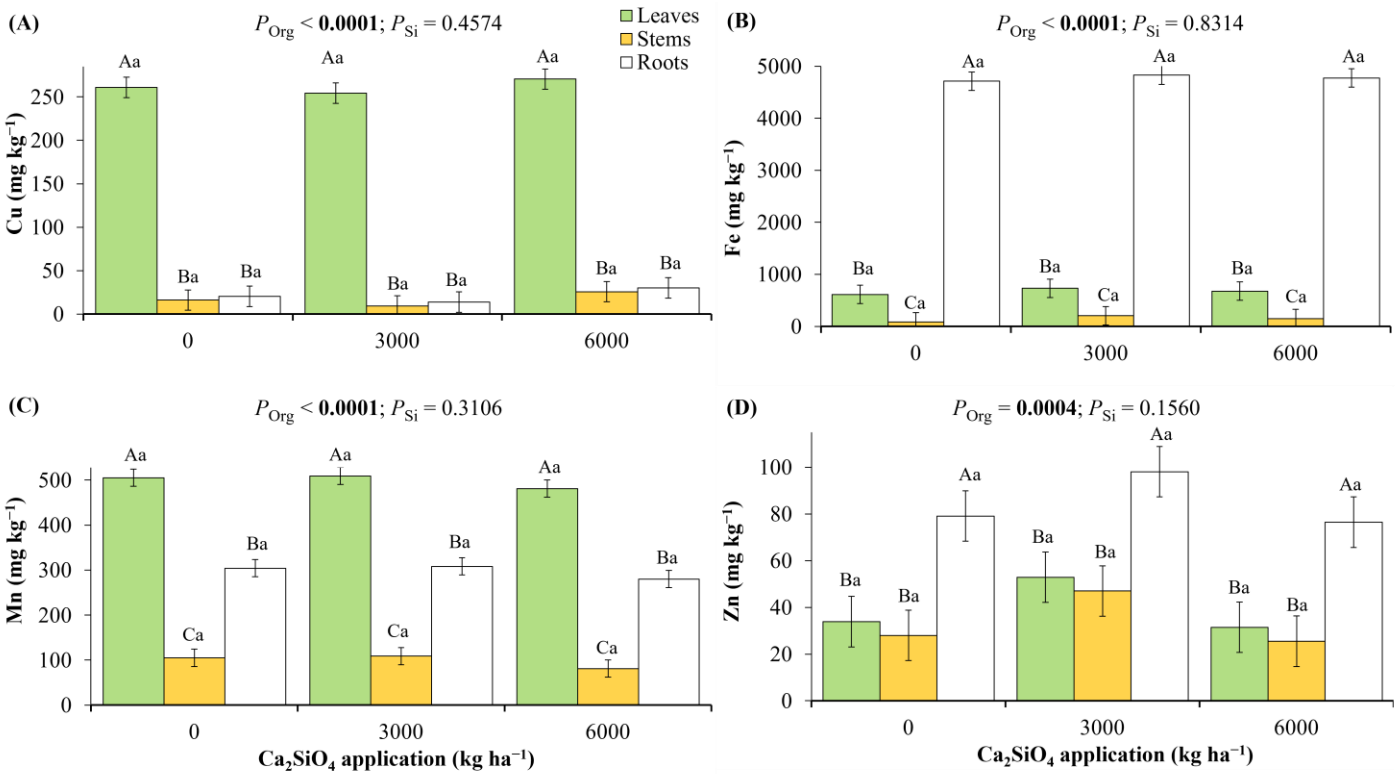

Figure S2. Variations in micronutrients: **A**) Cu, **B**) Fe, **C**) Mn, and **D**) Zn in leaves, stems, and roots of young coffee plants grown for six months under Ca_2SiO_4 supplying, corresponding to 0, 3000, and 6000 kg ha^{-1}. Estimated mean±SE, and *P*-values (bold when significant) are shown (n=3). Uppercase letters compare micronutrient concentrations among organs (Org) for each Ca_2SiO_4 (Si) treatment, while lowercase letters compare concentrations among Ca_2SiO_4 treatments for each plant organ.

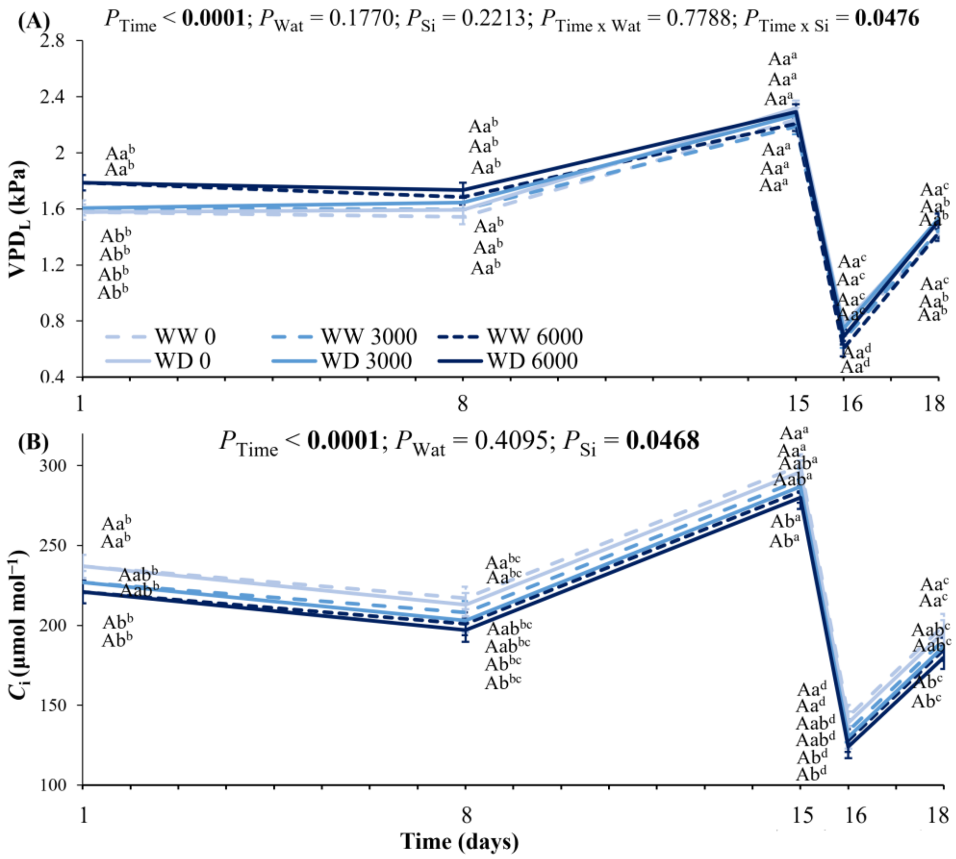

Figure S3. Variations in: **A**) leaf-to-air vapor pressure difference (VPD_L), and **B**) intercellular CO_2 concentration (C_i) of young coffee plants grown under Ca_2SiO_4 supplying, corresponding to 0, 3000, and 6000 kg ha^{-1} and subjected to two water regimes: well-watered (WW) and water deficit (WD) for 15 days, followed by re-watering and recovery period (days 16 and 18). Estimated mean±SE, and *P*-values (bold when significant) are shown (n=3). Uppercase letters compare water regimes (Wat) for each Ca_2SiO_4 (Si) treatment, and for each day of measurements; lowercase letters compare Ca_2SiO_4 treatments for each water treatment, and for each day of measurements; superscripted lowercase letters compare responses over time (Time) for each water regime and Ca_2SiO_4 treatment.

Disclaimer/Publisher's Note: The statements, opinions and data contained in all publications are solely those of the individual author(s) and contributor(s) and not of MDPI and/or the editor(s). MDPI and/or the editor(s) disclaim responsibility for any injury to people or property resulting from any ideas, methods, instructions or products referred to in the content.
